# Prevalence of and risk factors for increased serum levels of allergen-specific IgE in a population of Norwegian dogs

**DOI:** 10.1186/s13028-014-0081-z

**Published:** 2014-12-05

**Authors:** Annelin A Bjelland, Frederik L Dolva, Ane Nødtvedt, Bente K Sævik

**Affiliations:** Department of Companion Animal Clinical Sciences, Faculty of Veterinary Medicine and Biosciences, Norwegian University of Life Sciences, PO box 8146 Dep., NO-0033, Oslo, Norway; Department of Production Animal Clinical Sciences, Faculty of Veterinary Medicine and Biosciences, Norwegian University of Life Sciences, PO box 8146 Dep., NO-0033, Oslo, Norway; Department of Basic Sciences and Aquatic Medicine, Faculty of Veterinary Medicine and Biosciences, Norwegian University of Life Sciences, PO box 8146 Dep., NO-0033, Oslo, Norway

**Keywords:** Canine, Allergens, Immunoglobulins, Epidemiology, Serology, Allergy testing

## Abstract

**Background:**

The importance of different allergens in association with IgE production and canine atopic dermatitis (CAD) has been poorly studied and few studies exist on factors influencing allergen-specific IgE antibodies in serum. The aim of this cross-sectional study was to investigate the prevalence of elevated IgE levels to different environmental allergens in Norwegian dogs with a suspicion of CAD. The secondary aim was to identify risk factors associated with elevated serum levels of allergen-specific IgE.

**Results:**

The study sample consisted of serum from 1313 dogs of 161 different breeds. All samples were submitted for serologic IgE-testing (Fc epsilon R1 alpha-based ELISA) based on suspicion of CAD. Overall, 84.3% of the dogs had elevated IgE levels to one or more of the allergen(s). The predominant allergens amongst the positive results were the indoor allergens (*Acarus siro* 84.0%*, Dermatophagoides farinae* 80.2%*, Tyrophagus putrescentiae* 79.9%). Sheep sorrel was the most commonly encountered outdoor allergen (40.0%). Only 2.6% of the dogs with elevated IgE levels were positive to flea saliva.

The test results varied significantly depending on when the serum samples were taken. Samples taken during summer and autumn more often came out positive than samples taken during winter and spring. Geographical variations were also demonstrated. A greater proportion of females than males had positive test results, and more females than males tested positive to outdoor allergens. The mean age was significantly higher in the dogs testing positive than amongst the dogs testing negative. The allergen-specific IgE levels varied with breed. The boxer was the only breed with a significantly higher proportion of positive test results compared to the other breeds. Boxers also had a higher prevalence of elevated IgE levels to outdoor allergens, whereas the Rottweiler had a higher prevalence of elevated IgE levels to indoor allergens compared to the other breeds.

**Conclusions:**

IgE hypersensitivity was most often associated with indoor allergens. Outdoor allergens were of minor importance and IgE reactivity to flea saliva was rare. Breed differences in allergen-specific IgE levels were identified. Season of sampling, and the dogs’ geographical localisation, sex and age also affected the results of the IgE analysis.

## Background

Canine atopic dermatitis (CAD) is a commonly encountered disease in small animal practice. It is defined as “a genetically predisposed inflammatory and pruritic allergic skin disease with characteristic clinical features, most commonly associated with IgE antibodies to environmental allergens” [1]. Epidermal antigens, mite antigens, house dust, insect antigens, pollens and mould spores are examples of allergen groups associated with CAD [2].

Interaction between allergen-specific IgE antibodies and the relevant allergens is widely accepted as an important part of the pathogenesis of CAD, although it is known that CAD is a multifactorial disease that cannot be explained by IgE-mediated hypersensitivity alone [3,4]. Cutaneous antigen-presenting cells (Langerhans cells), T lymphocytes as well as many other cells and inflammatory mediators are all contributing to the disease process [4]. Also, a defective epidermal barrier function is thought to allow for the penetration of environmental allergens [4-6]. Many authors have investigated risk factors for the development of CAD. Geographical variations in the prevalence of the disease indicate that a dog's risk of developing CAD is influenced by geographical localisation. Climate, vegetation, life style, population density and pollution levels are all factors that might contribute to the varying occurrence of CAD in different regions [7-9]. Several studies document that certain breeds are overrepresented with the disease, indicating a genetic predisposition. However, the reported breeds vary between different publications. This could be explained by geographical and temporal differences in the genetic material of dog breeds, but might also be a result of different study populations [7,10,11]. There is no known sex predilection to CAD [12].

The initial diagnosis of CAD should be based on a set of clinical criteria; the currently favoured set was proposed by Favrot *et al.* [13]. It is also essential to exclude differential diagnoses, as well as carrying out an elimination diet trial to document whether food allergens are involved in the development of the disease [12,13]. After performing a proper work-up leading to the clinical diagnosis of CAD, IgE-based serologic or intradermal testing can be used to investigate whether or not the clinical signs are associated with allergen-specific IgE. Negative results to these tests occur in patients with atopic-like dermatitis (ALD), which is a disease with the same clinical features as CAD but with no detectable IgE response to allergens. Further, serologic and intradermal testing is useful to identify which allergens to tentatively reduce or eliminate from the patient's environment or which allergens to include in immunotherapy [12].

The influence of potential risk factors on serum levels of allergen-specific IgE has been poorly studied in the dog. It seems likely that geographical factors have an impact on the results of serologic IgE tests, as dogs in different regions are exposed to varying amounts of different allergens. Also, seasonal differences in allergen exposure presumably affect the serum levels of allergen-specific IgE [14]. Whether or not birth season influence allergen-specific IgE production has not been investigated, and studies on the association between birth season and CAD have shown conflicting results [7,15,16]. Breed differences in serum IgE levels have been documented in a few studies, indicating that the production of IgE antibodies is influenced by genetic factors [17,18]. There are conflicting results regarding the impact of a dog's age and sex on serum levels of IgE [17-19]. Vaccination regimens, parasitic disease and glucocorticoid administration are other factors that can influence serum IgE levels in dogs [14].

The prevalence of elevated IgE levels to specific allergens has been poorly studied in Norwegian dogs. Worldwide, only a few studies exist on factors that may increase the risk of developing elevated allergen-specific IgE levels in canine serum. Hence, the aim of the present study was to investigate the prevalence of elevated serum levels of IgE to different environmental allergens in serum samples from Norwegian dogs that underwent serologic allergen testing. A secondary aim was to identify risk factors associated with elevated serum levels of allergen-specific IgE.

## Materials and methods

### Study sample

In this cross-sectional study, the material was obtained from Dr. Baddaky AS (Skotterud, Norway), which is the only Norwegian enterprise to analyze allergen-specific IgE in animals. The full dataset consisted of serum samples from 1313 dogs, submitted to Dr. Baddaky AS for measurement of allergen-specific IgE (Heska Allercept® Serum IgE Test Nordic Panel) between March 2009 and September 2012. The serum samples were taken by veterinarians working in various geographic regions of Norway and were presumably submitted for IgE analysis based on suspicion of CAD.

Forms containing information from the referring veterinarians followed the serum samples and were registered in Dr. Baddaky's database. Information regarding when the samples were taken (the sample date), the postal codes of the submitting veterinarians, each dog's breed, sex and date of birth as well as the results from the IgE analysis were obtained from the database.

### IgE-testing

Heska Allercept® Serum IgE Test is an ELISA-based IgE analysis using the alpha chain of the human high affinity IgE receptor FcεRI as the IgE-specific reactant. The Nordic Panel includes indoor and outdoor allergens commonly occurring in Norway. The indoor allergens included in the Nordic Panel are the flour mite *Acarus siro*, the storage mite *Tyrophagus putrescentiae* and the house dust mite *Dermatophagoides farinae.* The outdoor allergens included are timothy grass (*Phleum pratense*), cocksfoot (*Dactylis glomerata*), blue grass annual (*Poa annua*), perennial rye grass (*Lolium perenne*), sheep sorrel (*Rumex* sp.), english plantain (*Plantago lanceolatum*), nettle (*Urtica dioica*), lambs quarter (*Chenopodium* sp.), mugwort (*Artemisia vulgaris*), alder (*Alnus* sp.), oak (*Quercus* sp.), birch (*Betula* sp.), meadow fescue (*Festuca elatior*), velvet grass (*Holcos lanatus*), redtop grass (*Agrostis* sp.), elm (*Ulmus* sp.), beech (*Fagus sylvatica*), hazel (*Corylus* sp.), sycamore (*Acer pseudoplatanus*) and ragweed (*Ambrosia* sp.). Flea saliva allergen is also included in the analysis.

The test results are given as a number on a continuous scale, ranging from 0 to 3000 (for some allergens 4000) enzyme linked immunosorbent assay (ELISA) absorbance units (EA units). This represents the amount of allergen-specific IgE measured in a serum sample. More information about the test procedure is available in the references [20-22].

A test result of 150 EA units or higher was considered positive. The dogs comprising the study sample were sorted according to their individual test results, providing the distribution of test results in the study sample.

### Risk factors

#### Breed

Based on the international breed standards (Federation Cynologique Internationale, FCI), the dogs were categorized by their respective breed, giving a total of 161 different breeds. All mixed breeds were collectively called mixed breed.

#### Sex

Each dog was categorized as either male or female, based on the information from the submitting veterinarians. Neutered individuals were not separated into isolated categories, because of uncertainty regarding the completeness of this information. Routine spay/neutering of healthy dogs is not commonly practiced in Norway.

#### Age

Each dog's age in months was calculated by subtracting the birth date from the sample date. The dogs were categorized into ten age categories: 0–6 months, >6 months −1 year, >1-2 years, >2-3 years, >3-4 years, >4-5 years, >5-6 years, >6-7 years, >7-8 years and >8 years.

#### Season

Each dog's birth season was defined based on the birth date: Spring (March, April, May), summer (June, July, August), autumn (September, October, November) and winter (December, January, February). Season of sampling was categorized in the same manner, depending on the sample date.

#### Geographical region

The postal codes of the submitting veterinarians were used to identify the geographical distribution of the study sample. Counties were generated from the postal codes [23]. The 19 counties of Norway were then sorted into seven regions based on a national standard of regional division [24]: Oslo/Akershus (Oslo, Akershus), Hedmark/Oppland (Hedmark, Oppland), Southeast (Østfold, Buskerud, Vestfold, Telemark), Agder/Rogaland (Aust-Agder, Vest-Agder, Rogaland), Northwest (Hordaland, Sogn og Fjordane, Møre og Romsdal), Trøndelag (Sør-Trøndelag, Nord-Trøndelag) and North (Nordland, Troms, Finnmark).

### Statistical analysis

The data was transferred into a database created in Excel® (Microsoft® Excel® for Mac 2011, Version 14.3.8.). The dataset was sorted in pivot tables, and all tables and graphs were created using a filtering function in Excel®.

The mean age of the dogs in the study sample was estimated with a 95% confidence interval (CI) using Epitools [25]. Non-overlapping confidence intervals for the mean age in different groups were seen as evidence of a statistically significant age difference.

The relationship between the risk factors sex, birth season, sample season and geographical region and the results from the allergen-specific IgE test (+/−) were tabulated and analyzed using a chi-square test. The chi-square test was performed with a 95% confidence limit using Epitools [26]. A *P*-value of <0.05 was considered statistically significant.

Statistical analysis of breed as a risk factor was performed using the software package Stata SE 12 (Stata statistical software, College Station, Texas, USA). The 95% confidence limit for the proportion of dogs with positive test results within each breed was calculated. Deviation from the “average breed” (pooled test-result across all breeds) was evaluated based on non-overlapping confidence intervals. The breeds represented with less than 10 individuals in the study sample were excluded from the analysis of breed effect.

## Results

### Study sample

The study sample consisted of serum from 1313 dogs. A total of 161 breeds were represented. Besides mixed breed (123 dogs; 9.4%), the German shepherd dog was the most numerous breed (102 dogs; 7.8%). The sex distribution was similar, with 673 males (51.3%) and 640 females (48.7%). The age of 106 of the 1313 individuals could not be calculated due to lack of information. The remaining 1207 dogs had a mean age of 3.3 years (39.2 months). As with age, the birth season of 106 dogs could not be defined due to lack of information. Amongst the remaining 1207 dogs, a slight majority were born in the spring (374 dogs; 31.0%) and slightly fewer were born in the winter (257 dogs; 21.3%). 285 dogs (23.6%) were born in the autumn and 291 dogs (24.1%) were born in the summer. The season of IgE analysis showed more variation. Most serum samples were taken in the summer (462 of the 1313 samples; 35.2%) and autumn (400 samples; 30.5%), while only 181 samples (13.8%) were taken during the winter months. 270 samples (20.6%) were taken in the spring. A majority of the 1313 samples were sent from the southern parts of Norway, with a total of 1174 (89.4%) coming from regions south of Trøndelag.

### IgE test results

1107 of the 1313 dogs (84.3%) had IgE levels of 150 EA units or higher (i.e. positive results) for one or more of the allergen(s) included in the test (T+). 595 dogs (45.3%) had a positive reaction against at least one outdoor allergen (O+), while 1018 dogs (77.5%) reacted positive against at least one indoor allergen (I+). Most dogs that tested positive had elevated IgE levels for both outdoor and indoor allergens (O+ and I+, 506 of the 1107 dogs, 45.7%) or indoor allergens alone (O- and I+, 46.3%). Only 89 of the dogs with positive results (8.0%) were positive to outdoor allergens alone (O+ and I*-*).

The distribution of positive results to each allergen is presented in Table 1. The predominant allergens amongst the positive results were

**Table 1:** **The number and proportion of elevated serum levels of allergen-specific IgE to each allergen in a database of test results from serologic allergen-specific IgE-testing in Norwegian dogs**

**Allergen**	**Number**	**Proportion (%)**
**Outdoor allergens**		
Sheep sorrel	443	40.0
Redtop grass	415	37.5
Cocksfoot	413	37.3
Meadow fescue	405	36.6
Beech	401	36.2
Velvet grass	388	35.0
Perennial rye grass	385	34.8
Timothy grass	368	33.2
Blue grass anual	334	30.2
Ragweed	288	26.0
English plantain	278	25.1
Elm	275	24.8
Lambs quarter	261	23.6
Hazel	244	22.0
Alder	232	21.0
Oak	226	20.4
Sycamore	219	19.8
Mugwort	219	19.8
Birch	183	16.5
Nettle	136	12.3
**Indoor allergens**		
*Acarus siro*	930	84.0
*Dermatophagoides farinae*	888	80.2
*Tyrophagus putrescentiae*	885	79.9
**Flea saliva**	29	2.6

Number of samples with IgE >150 *EA* units, and the corresponding proportion (%) of all samples with positive results.

*A. siro* (930/1107; 84.0%), *D. farinae* (888/1107; 80.2%) and *T. putrescentiae* (885/1107; 79.9%). Sheep sorrel was the most commonly encountered outdoor allergen, with 443 of the 1107 dogs (40.0%) testing positive. Elevated IgE levels to flea saliva was by far the least common result in the study sample, with 29 of the 1107 dogs (2.6%) testing positive. Only two dogs tested positive to flea saliva alone.

### Risk factors

#### Sex

The association between sex and results of the IgE analysis are shown in Table 2. A greater proportion of females than males had positive test results (T+; *P* = 0.035), and more females tested positive to outdoor allergens than did males (O+; *P* < 0.0001). No statistically significant difference between sexes was detected for indoor allergens.

**Table 2:** **The distribution of test results in males and females in a database of test results from serologic allergen-specific IgE-testing in Norwegian dogs**

**Test result**	**Male**	**Female**	***P*** **-value**
	**Number (%)**	**Number (%)**	
**Overall (any allergen)**			
T+	553 (82.2)	554 (86.6)	0.0347
T-	120 (17.8)	86 (13.4)	
**Outdoor allergens**			
O+	269 (40.0)	326 (50.9)	<0.0001
O-	404 (60.0)	314 (49.1)	
**Indoor allergens**			
I+	508 (75.5)	510 (79.7)	0.0787
I-	165 (24.5)	130 (20.3)	

A *P*-value of <0.05 implies a statistically significant difference between the sexes based on a chi-square test.

#### Age

The age was known for 1022 of the 1107 dogs testing positive (T+). Only one of the positive individuals (0.1%) was younger than six months, while 42 dogs (4.1%) were between six and twelve months of age. A majority of the dogs with positive results were between one and four years old (679 dogs; 66.4 %). The age category >1-2 years was the most numerous (272 dogs; 26.6%). The number of positive dogs then decreased with advancing age.

The mean age was significantly higher in the dogs testing positive (T+; mean age 39.6 months; 95% CI: 39.3-39.9 months) than amongst the dogs with negative results (T-; mean age 37.3 months; 95% CI: 36.6-38.1). The same significant variation was seen for outdoor allergens (O+; mean age 40.6 months; 95% CI: 40.2-41.1 and O-; mean age 38.1 months; 95% CI: 37.7-38.5), while the mean age of the dogs testing positive and negative to indoor allergens, respectively, was not significantly different (I+; mean age 37.7 months; 95% CI: 37.1-38.3 and *I-*; mean age 38.1 months; 37.7-38.5).

#### Birth season

No statistically significant association could be found between the dog's birth season and results of the IgE analysis. Amongst the dogs born during spring, 318 (85.0%) had positive test results (T*+*), while 56 dogs (15.0%) were negative to all allergens (T*-*). 253 of the dogs born in the summer (86.9%), 239 of the dogs born in the autumn (83.9%) and 212 of the dogs born in the winter (82.5%) had positive test results (T*+*). The corresponding *P*-value, as calculated by chi-squared test, was 0.5166. The relationship between birth season and results to outdoor and indoor allergens (O+/O- and I+/I-) also was insignificant (*P*-value 0.7124 and 0.3303, respectively).

#### Sample season

The test results varied significantly depending on when the serum samples were taken (Table 3). Samples taken during summer and autumn more often came out positive (T*+*) than samples taken during winter and spring. The same pattern was identified in terms of serum IgE levels to outdoor and indoor allergens, respectively.

**Table 3:** **The distribution of test results in samples taken during different seasons of the year in a database of test results from serologic allergen-specific IgE-testing in Norwegian dogs**

**Test result**	**Spring**	**Summer**	**Autumn**	**Winter**	***P*** **-value**
	**Number (%)**	**Number (%)**	**Number (%)**	**Number (%)**	
**Overall (any allergen)**					
T+	214 (79.3)	399 (86.4)	350 (87.5)	144 (79.6)	0.005
T-	56 (20.7)	63 (13.6)	50 (12.5)	37 (20.4)	
**Outdoor allergens**					
O+	106 (39.3)	220 (47.6)	199 (49.8)	70 (38.7)	0.0098
O-	164 (60.7)	242 (52.4)	201 (50.3)	111 (61.3)	
**Indoor allergens**					
I+	188 (69.6)	373 (80.7)	319 (79.8)	138 (76.2)	0.0033
I-	82 (30.4)	89 (19.3)	81 (20.2)	43 (23.8)	

A *P*-value of <0.05 implies a statistically significant difference among the different seasons based on a chi-square test.

#### Geographical region

The relationship between the dog’s geographical region and results of the IgE analysis were statistically significant. The region North had the lowest proportion of positive results (T+; 76% of all samples coming from this region), while the southern regions Agder/Rogaland and Southeast had the highest proportion of positive results (90.4% and 91.1%, respectively). The proportion of positive test results amongst the remaining regions varied between 80.2% (Northwest) and 84.4% (Trøndelag). The *P*-value for the geographical distribution of positive and negative test results (T+/T-), as calculated by chi-squared test, was 0.0008.

Oslo/Akershus had the highest proportion of dogs testing positive to outdoor allergens (O+; 52.8% of all samples coming from this region), with the southern regions Agder/Rogaland and Southeast having the second highest proportion of positive results to outdoor allergens (51.1% and 51.1%, respectively). Northwest had the lowest proportion of positive results to outdoor allergens (32.6%). The remaining regions had the following distribution of positive results to outdoor allergens (O+): North 40.0%, Hedmark/Oppland 42.6%, Trøndelag 45.3%. The *P*-value for the geographical distribution of positive and negative results to outdoor allergens (O+/O*-*), was <0.0001.

The same *P*-value (<0.0001) was calculated for the geographical distribution of positive and negative results to indoor allergens (I+/I-). Agder/Rogaland had the highest proportion of dogs testing positive to indoor allergens (I+; 87.5% of all samples coming from this region). The region North had the lowest proportion of positive results to indoor allergens (60.0%). The proportion of positive results to indoor allergens amongst the remaining regions varied between 74.1% (Oslo/Akershus) and 83.7% (Southeast).

#### Breed

Out of the 161 breeds registered in the study sample, 30 breeds were represented with at least 10 individuals. A total of 915 dogs were represented amongst these 30 breeds. The distribution of positive test results within these breeds is shown in the Tables 4, 5 and 6. The boxer was the only breed with a significantly higher proportion of positive test results compared to the overall average across breeds (T+; 58 out of the 59 boxers; 98%). Pugs and Jack Russell terriers, on the other hand, had a significantly lower proportion of positive test results (T+; 59% and 66%, respectively). The boxer also was the breed with the highest proportion of positive results to outdoor allergens (O+; 73%), while the flat-coated retriever and the German shepherd dog had the lowest proportion of dogs testing positive to these allergens (O+; 17% and 29%, respectively). The highest proportion of positive results to indoor allergens was found in the Rottweiler (I+; 98%), while the English setter had the lowest proportion of positive results to these allergens (I+; 55%).

**Table 4:** **The breed distribution of elevated serum IgE levels to all allergens**

**Breed**	**Number of results in the study sample**	**Number T+**	**Proportion T** ***+***	**95% CI for proportion T** ***+***
Total (all breeds)	915	789	0.86	0.84 - 0.89
American cocker spaniel	17	15	0.88	0.64 - 0.99
American Staffordshire terrier	10	8	0.8	0.44 - 0.97
Bernese mountain dog	11	10	0.91	0.59 - 1.00
Bichon frisé	26	22	0.85	0.65 - 0.96
Mixed breed	123	112	0.91	0.85 - 0.95
Dogue de Bordeaux	10	7	0.7	0.35 - 0.93
Boxer	59	58	0.98	0.91 - 1.00
Bull terrier	13	10	0.77	0.46 - 0.95
Cavalier King Charles spaniel	15	10	0.67	0.38 - 0.88
Chinese crested dog	10	9	0.9	0.55 - 1.00
English cocker spaniel	16	12	0.75	0.48 - 0.93
Dachshond short hair	10	10	1	0.69 - 1.00*
Dachshund wire-hair	10	10	1	0.69 - 1.00*
Dalmatian	12	11	0.92	0.62 - 1.00
Bulldog	16	13	0.81	0.54 - 0.96
English setter	38	27	0.71	0.54 - 0.85
Flat-coated retriever	24	19	0.79	0.59 - 0.93
French bulldog	25	22	0.88	0.69 - 0.97
Golden retriever	56	51	0.91	0.80 - 0.97
Gordon setter	23	22	0.96	0.78 - 1.00
Soft-coated wheaten terrier	16	13	0.81	0.54 - 0.96
Jack Russell terrier	29	19	0.66	0.47 - 0.82
Labrador retriever	83	74	0.89	0.80 - 0.95
Pug	17	10	0.59	0.33 - 0.82
Newfoundland	14	13	0.93	0.66 - 1.00
Nova Scotia duck tolling retriever	12	12	1	0.74 - 1.00*
Rottweiler	43	42	0.98	0.88 - 1.00
German shepherd dog	102	82	0.8	0.71 - 0.86
Staffordshire bull terrier	61	53	0.87	0.76 - 0.94
Tibetan spaniel	14	13	0.93	0.66 - 0.88

*One sided 97.5% CI.

Number of samples, number of test positive samples (T+), proportion of T+ and 95% confidence interval (CI) for the proportion of T+ of all samples from the relevant breed.

**Table 5:** **The breed distribution of elevated serum IgE levels to outdoor allergens**

**Breed**	**Number of results in study sample**	**Number O+**	**Proportion O+**	**95% CI for proportion O+**
Total (all breeds)	915	422	0.46	0.43 - 0.49
American cocker spaniel	17	8	0.47	0.23 - 0.72
American Staffordshire terrier	10	5	0.5	0.19 - 0.81
Bernese mountain dog	11	3	0.27	0.06 - 0.61
Bichon frisé	26	12	0.46	0.27 - 0.67
Mixed breed	123	69	0.56	0.47 - 0.65
Dogue de Bordeaux	10	4	0.4	0.12 - 0.74
Boxer	59	43	0.73	0.60 - 0.84
Bull terrier	13	3	0.23	0.05 - 0.54
Cavalier King Charles spaniel	15	5	0.33	0.12 - 0.62
Chinese crested Dog	10	5	0.5	0.19 - 0.81
English cocker spaniel	16	4	0.25	0.07 - 0.52
Dachshund short hair	10	5	0.5	0.19 - 0.81
Dachshund wire-hair	10	6	0.6	0.26 - 0.88
Dalmatian	12	8	0.67	0.35 - 0.90
Bulldog	16	4	0.25	0.07 - 0.52
English setter	38	23	0.61	0.43 - 0.76
Flat-coated retriever	24	4	0.17	0.05 - 0.37
French bulldog	25	11	0.44	0.24 - 0.65
Golden retriever	56	28	0.5	0.36 - 0.64
Gordon setter	23	16	0.7	0.47 - 0.87
Soft-coated wheaten terrier	16	8	0.5	0.25 - 0.75
Jack Russell terrier	29	10	0.34	0.18 - 0.54
Labrador retriever	83	28	0.34	0.24 - 0.45
Pug	17	4	0.24	0.07 - 0.50
Newfoundland	14	4	0.29	0.08 - 0.58
Nova Scotia duck tolling retriever	12	8	0.67	0.35 - 0.90
Rottweiler	43	23	0.53	0.38 - 0.69
German shepherd dog	102	30	0.29	0.21 - 0.39
Staffordshire bull terrier	61	35	0.57	0.44 - 0.70
Tibetan spaniel	14	6	0.43	0.18 - 0.71

Number of samples, number of test positive samples to outdoor allergens (O+), proportion of O+ and 95% confidence interval (CI) for the proportion O+ of all samples from the relevant breed.

**Table 6:** **The breed distribution of elevated serum IgE levels to indoor allergens**

**Breed**	**Number of results in study sample**	**Number I+**	**Proportion I+**	**95% CI for proportion I+**
Total (all breeds)	915	717	0.78	0.76 - 0.81
American cocker spaniel	17	13	0.76	0.50 - 0.93
American Staffordshire terrier	10	8	0.8	0.44 - 0.97
Bernese mountain dog	11	9	0.82	0.48 - 0.98
Bichon frisé	26	19	0.73	0.52 - 0.88
Mixed breed	123	98	0.8	0.71 - 0.86
Dogue de Bordeaux	10	7	0.7	0.35 - 0.93
Boxer	59	49	0.83	0.71 - 0.92
Bull terrier	13	10	0.77	0.46 - 0.95
Cavalier King Charles spaniel	15	10	0.67	0.38 - 0.88
Chinese crested dog	10	7	0.7	0.35 - 0.93
English cocker spaniel	16	11	0.69	0.41 - 0.89
Dachshund short hair	10	9	0.9	0.55 - 1.00
Dachshund wire-hair	10	10	1	0.69 - 1.00*
Dalmatian	12	10	0.83	0.52 - 0.98
Bulldog	16	12	0.75	0.48 - 0.93
English setter	38	21	0.55	0.38 - 0.71
Flat-coated retriever	24	19	0.79	0.58 - 0.93
French bulldog	25	18	0.72	0.51 - 0.88
Golden retriever	56	49	0.88	0.76 - 0.95
Gordon setter	23	21	0.91	0.72 - 0.99
Soft-coated wheaten terrier	16	11	0.69	0.41 - 0.89
Jack Russell terrier	29	17	0.59	0.39 - 0.76
Labrador retriever	83	73	0.88	0.79 - 0.94
Pug	17	9	0.53	0.28 - 0.77
Newfoundland	14	13	0.93	0.66 - 1.00
Nova Scotia duck tolling retriever	12	8	0.67	0.35 - 0.90
Rottweiler	43	42	0.98	0.88 - 1.00
German shepherd dog	102	77	0.75	0.66 - 0.83
Staffordshire bull terrier	61	46	0.75	0.63 - 0.86
Tibetan spaniel	14	11	0.79	0.49 - 0.95

*One sided 97.5% CI.

Number of samples, number of test positive samples to indoor allergens (I+), proportion of I+ and 95% confidence interval (CI) for the proportion I+ of all samples from the relevant breed.

## Discussion

### Prevalence of elevated serum IgE levels to environmental allergens

A large majority (84.3%) of the dogs in the present study had positive results to one or more of the allergens included in the Nordic panel serum IgE test. The actual prevalence of elevated IgE levels in the general dog population is not known [9], although it is reasonable to believe that the prevalence is lower than the results from this study. The high proportion of positive results might be caused by a combination of factors. Firstly, the serum samples were presumably submitted for IgE analysis based on suspicion of CAD. However, the diagnostic work-up performed by the submitting veterinarians was not investigated in this study. Provided that the dogs have been correctly diagnosed with CAD before the IgE analysis, the negative testing individuals could represent the diagnosis of atopic-like dermatitis (ALD). Secondly, Dr. Baddaky AS recommends performing Allercept® Screening test to measure total IgE levels to different allergen groups (grass pollen, tree pollen and indoor allergens) before embarking upon the allergen-specific assays. The Nordic Panel is recommended following positive results to both indoor and outdoor allergens on the screening test [27]. Hence, the study sample was not randomly selected but represents a subset of dogs in which veterinarians have suspected hypersensitivity to both indoor and outdoor allergens, and no conclusion can be made about the prevalence of elevated allergen-specific IgE levels in the general Norwegian dog population from this study. However, the results can probably be extrapolated to other populations of dogs subjected to allergy testing in other geographic regions.

The predominant allergens amongst the positive results were the indoor allergens: *A. siro* (84.0%), *D. farinae* (80.2%) and *T. putrescentiae* (79.9%). It should be noted that the Nordic Panel only includes a subset of selected indoor allergens, and some of these might be false positive results as former studies have documented cross-reactivity amongst the mites [28]. Most dogs testing positive had elevated IgE levels to both indoor and outdoor allergens (45.7%) or indoor allergens alone (46.3%). Only 8.0% of the dogs with positive results were positive to outdoor allergens alone. This agrees well with other studies from the Nordic countries. In a report based on intradermal testing, *D. farinae* was found to be the most important allergen amongst Norwegian dogs with CAD [29]. In a study on a group of 62 atopic dogs from the Nordic countries (Denmark, Finland, Norway and Sweden), 93.5% had positive results to house dust mites (*D. farinae* and/or *D. pteronyssinus*). Only 16.1% were positive to pollen allergens in the same study, and none of the dogs reacted to pollen allergens alone. This study was largely based on intradermal testing [30]. Unfortunately, one cannot directly compare our results with these former studies, as there is only partial correlation between serologic and intradermal test results [14]. Several international studies based on serological IgE tests exist, however, the results differ markedly. The lack of a universal standardization of allergen extracts and test methodologies, as well as geographical differences in exposure to different allergens are all factors that could explain the heterogeneous results between the different publications. Nevertheless, one can see a general trend from the existing studies. *Dermatophagoides* spp. seems to be a major allergen in many parts of the world, including Europe. Pollen allergens on the other hand, appear to be important in the USA, but of less importance in Europe [2,14]. The present study implies that Norwegian dogs are more prone to develop hypersensitivity to indoor allergens than pollen allergens, similar to the trend reported in other parts of Europe [2].

Elevated IgE levels to flea saliva was by far the least common result in the study sample (2.6%). This is not surprising, because the flea burden in Norway is relatively low and dogs in areas with low numbers of fleas have a low prevalence of hypersensitivity to flea allergens [31].

### Risk factors

In this study, the dogs’ sex, age, breed and geographical region influenced serum levels of allergen-specific IgE. Sampling season also affected the test results, whereas birth season had no significant influence on the IgE levels.

The results from previous studies are ambiguous regarding whether or not there is a sex predilection to elevated serum levels of allergen-specific IgE in dogs [17-19]. In the present study, female dogs had a significantly higher proportion of positive test results than did males. Furthermore, more female than male dogs tested positive to outdoor allergens, whereas no statistically significant difference between sexes was detected for indoor allergens. In conclusion, our results show that female dogs in Norway have a higher risk of developing elevated IgE levels than male dogs, particularly to outdoor allergens. Assuming that IgE antibodies play an essential role in the pathogenesis of CAD and that the dogs have been correctly diagnosed with CAD before the IgE analysis, the results of this study contradict the general consensus that there is no sex predilection to the disease. However, one must keep in mind that neutered and intact individuals were not separated in the present study.

In the current study, the group of dogs with elevated IgE levels to one or more of the allergens had a higher mean age (39.6 months) than the group of dogs with non-elevated IgE levels (37.3 months). Dogs with positive results to outdoor allergens had a higher mean age (40.6 months) than dogs testing negative to the same group of allergens (38.1 months). The age differences were statistically significant, as documented by non-overlapping confidence intervals. This implies that the production of allergen-specific IgE, particularly to outdoor allergens, varies with age. On the other hand, no significant variation was found in mean age between dogs testing positive and negative to indoor allergens (37.7 and 38.1 months, respectively). Another interesting finding, however, is that dogs with positive results to outdoor allergens had a higher mean age than dogs testing positive to indoor allergens. This could imply that the production of IgE to outdoor allergens is dependent on allergen exposure over a prolonged period of time, compared to the production of IgE to indoor allergens. One could also speculate that Norwegian dogs are more exposed to indoor allergens than outdoor allergens, making elevated IgE levels to indoor allergens appear earlier. The relationship between dogs’ age and serum levels of IgE has been poorly studied, and the existing reports are conflicting. A study on healthy beagles found that total serum IgE levels varied with age [19], whereas some of the same authors in another publication concluded that age did not have any influence [18]. In a study on atopic and non-atopic Labrador- and golden retrievers, no association could be found between age and IgE levels, except that the dogs testing positive to flea saliva were older than the dogs testing negative to the same allergen [17].

The present study documents statistically significant geographical variations in canine serum levels of allergen-specific IgE. Several underlying factors could explain this variation. Climate differences might be a factor involved. The region with the lowest proportion of positive results, North, has a lower level of average annual rainfall and lower humidity compared to the other regions [32]. This leads to less suitable conditions for the mites [33], which were found to be the most important allergens in the present study sample. Also, there is less exposure to pollen allergens in North than in other parts of Norway [34]. Population density is another factor that could play a role; North is more sparsely populated than the other regions. To our knowledge, no previous studies on geographical variation in canine allergen-specific IgE have been performed. However, several studies on CAD and atopic diseases in people support the findings in the present study. Average annual rainfall was positively associated with the incidence rate of CAD in a Swedish study, and increasing human population density has been associated with increased occurrence of atopic diseases in both dogs and people [8,9,15]. Pollution levels, lifestyle, vaccination routines and parasite control regimes are other geographically varying factors which could contribute to the geographical differences seen in the present and previous studies [8,9].

Significantly higher proportions of the serum samples taken during summer and autumn were positive on the IgE test than samples taken during winter and spring in the present study. This pattern was found for the test overall (T+), as well as for the outdoor (O+) and indoor allergens (I+). The literature is limited in terms of seasonal variations in allergen-specific IgE levels in dogs, but season of the year has been found to influence the results of serum IgE tests in people [14]. One could speculate that these findings are caused by seasonal differences in allergen exposure. In Norway, the highest levels of pollen allergens are seen during the spring and summer months [34]. In humans, the IgE levels rise with pollen exposure, peaking approximately four weeks after the exposure, and then declining [14]. If the same is true for dogs, this delay could explain why the proportion of positive results is higher during summer and autumn than during winter and spring. Further, the relative humidity is higher during the warm season than during winter in Norway. Higher humidity levels leads to more suitable conditions for the house dust mite [35], which could explain the results in terms of indoor allergens.

The dogs' birth season did not affect the results of the IgE analysis in this study. To the authors’ knowledge, no other published studies have investigated the association between birth season and allergen-specific IgE levels, and results from studies on CAD are conflicting [7,15,16].

The present study documents breed differences in serum levels of allergen-specific IgE. There was a significantly higher proportion of elevated IgE levels (T+) amongst the boxers than amongst the other breeds in the study sample, indicating that boxers may be more prone to produce IgE antibodies in response to environmental allergens than other breeds. Pugs and Jack Russell terriers seem to be at a lower risk to develop IgE hypersensitivity, as these breeds had a significantly lower proportion of positive results on the IgE test compared to the other breeds represented. Provided that the history of the dogs in the study sample matches the clinical diagnosis of CAD, one could speculate whether pugs and Jack Russell terriers are overrepresented with ALD compared to other breeds.

Further, the present study shows that the propensity to produce IgE antibodies to different allergen groups varies with breed. On the basis of the current results, it seems like the boxer are predisposed to producing IgE antibodies to outdoor allergens (O+), while the Rottweiler are predisposed to developing IgE hypersensitivity towards indoor allergens (I+). This is an interesting finding, as breed differences in allergen-specific IgE reactivity previously have been poorly studied in the dog; most studies on the subject have evaluated breed differences in total serum IgE levels. Also, several studies exist on breed predispositions to the disease CAD. Both total IgE levels and the occurrence of CAD have been documented to vary with breed [7,10,11,17,18], and the results from the present study indicate that there also is breed variations in terms of which allergens dogs react to.

## Conclusions

IgE hypersensitivity among Norwegian dogs with suspected CAD was most often associated with indoor allergens. Outdoor allergens were of minor importance and IgE reactivity to flea saliva was rare. Serum levels of allergen-specific IgE varied with sex, age, geographical localisation, and sampling season. Breed differences in allergen-specific IgE levels were documented. Relative to the other breeds represented in the study sample, the boxer had a higher prevalence of elevated IgE levels to outdoor allergens, while the Rottweiler had a higher prevalence of elevated IgE levels to indoor allergens.
